# Influence of nitrogen and phosphorous on the growth and root morphology of *Acer mono*

**DOI:** 10.1371/journal.pone.0171321

**Published:** 2017-02-24

**Authors:** Muhammad Razaq, Peng Zhang, Hai-long Shen, Salahuddin

**Affiliations:** 1 School of Forestry, Northeast Forest University, Harbin, China; 2 Agricultural Research Institute, Mingora, Pakistan; University of Vigo, SPAIN

## Abstract

Nitrogen and phosphorous are critical determinants of plant growth and productivity, and both plant growth and root morphology are important parameters for evaluating the effects of supplied nutrients. Previous work has shown that the growth of *Acer mono* seedlings is retarded under nursery conditions; we applied different levels of N (0, 5, 10, and 15 g plant^-1^) and P (0, 4, 6 and 8 g plant^-1^) fertilizer to investigate the effects of fertilization on the growth and root morphology of four-year-old seedlings in the field. Our results indicated that both N and P application significantly affected plant height, root collar diameter, chlorophyll content, and root morphology. Among the nutrient levels, 10 g N and 8 g P were found to yield maximum growth, and the maximum values of plant height, root collar diameter, chlorophyll content, and root morphology were obtained when 10 g N and 8 g P were used together. Therefore, the present study demonstrates that optimum levels of N and P can be used to improve seedling health and growth during the nursery period.

## Introduction

The aesthetic and economic values of *Acer mono* Maxim L. (Aceraceae) make it an important species for afforestation, gardening, and road plantings. The species is widely distributed in the Yangtze River basin of north and northeast China [1]. Nursery practices, such as sowing, seedbed density, pruning, and fertilization, are usually standardized for individual plant species, in order to produce high-quality seedlings [2]. Fertilizer application is widely used in nurseries to improve plant vigor and productivity [3]; however, fertilization can improve plant growth by either increasing soil resources or by enhancing the ability of seedlings to garner resources [4] by modifying soil pH [5]. As a result, plants increase their rate of photosynthesis, stem diameter, height, basal area, and volume [6]. Deciduous and evergreen hardwood species have different nutrient requirements, and deciduous species have been reported to require 50% more N than conifers, such as pines [7]. Therefore, the production of deciduous and pine seedlings have different nutrition and management requirements [8].

Of the necessary nutritional elements, N is required in the largest quantities, and its availability and internal concentration affect the partitioning of biomass between roots and shoots [9]. The amount and timing of N application can also alter plant morphology, nutrient availability, and net photosynthesis [10]. For example, Harper [11] reported that N supplementation is required to maximize seedling biomass during initial nursery stages of growth, even for some legume species, and Costa *et al*. [12] reported that root length and root surface area were increased under intermediate N levels and that root growth was reduced under both higher and lower fertilization levels. However, high N availability and its concomitant affect root and shoot biomass production [13]. Phosphorus is considered a primary nutrient for plant growth [14] and is needed to sustain optimum plant production and quality [15]. The element is essential for cell division, reproduction, and plant metabolism; moreover, its role is related to the acquisition, storage, and use of energy [16]. In addition, P plays an important role in lateral root morphology and root branching [17] and influences not only root development, but also the availability of nutrients [18]. Therefore, plants have developed various strategies for obtaining optimum P from soils, including increases in root surface area, specific root length (SRL), and root-shoot ratio [19,20].

Both N and P are important nutrients for ecosystem structure, processes, and function, since their availability limits the production of plant biomass and growth [21]. For example, the combined application of N and P increases root surface area, root length, and root-shoot mass [22], and in *Arabidopsis* plant species, different nutrient levels have been shown to influence both root length and branching plasticity [23].

In the last few decades, the application of fertilizer in forest nurseries has attracted increasing attention throughout the world, as a result of increased demand for fiber, wood [24], and CO_2_ offsets [25]. These demands can be satisfied through the production of healthy seedlings, which would ultimately increase plant biomass production; however, this requires the proper diagnosis of specific limiting factors [26]. In fact, the amounts of fertilizers used in forest seedling production are relatively lower than the amounts used in agriculture plant nurseries [27]. Many factors influence the effectiveness of nutrient application on seedling growth. In particular, fertilizer type and amount affect the growth of plant seedlings [28].

Since growth of *A*. *mono* seedlings is reportedly retarded under nursery conditions [29], species-specific combinations of N and P may be needed to ensure healthy seedling growth. Therefore, the present study was designed to determine the effects of N and P on *A*. *mono* seedling growth, with a specific focus on root morphology (i.e., root length, root diameter, SRL), and to establish a standard for N and P application in the production of *A*. *mono* seedlings.

## Materials and methods

### Study site and soil collection

This experiment was conducted at Maoershan Forest Research Station (127°′‒127°′E, 45°23′‒45°26′ N, 390 m above sea level) of Northeast Forestry University, in Heilongjiang, China. The site has a cold, continental monsoon climate, with an average annual air temperature of 2.8°C. Average temperatures in January and July are -19.6°C and 20.9°C, respectively, and the annual average humidity, annual precipitation, and annual evaporation are 70%, 723.8 mm, and 1094 mm, respectively. The frost-free period usually lasts from 120 to 140 d, and the soil is mostly Hap-Boric Luvisol [30], which was described previously by Wang *et al*. [31].

Before starting experiment ten soil samples were collected from the experimental field at 0–20 cm soil depth and were thoroughly mixed to make a representative composite soil sample that had a total N, P, and K content of 3.98 g kg^-1^, 820.8 mg kg^-1^, and 14 g kg^-1^, respectively, and an available N, P, and K content of 4, 7.23, and 176 mg kg^-1^. The total N was determined by an elemental analyzer (vario MACRO cube; Elementar, Hanau, Germany). The available nitrogen was measured by alkali solution diffusion method and total P and available P by flow injection analyzer method (Seal Autoanalyzer 3, Seal Analytical, Norderstedt, Germany). Total K and available K were determined by the flame photometer (FP640, Shanghai, China). The soil test was performed in State Forestry Administration Key Laboratory of forest resources development in Northeast China.

### Plant materials and fertilization treatments

The seeds of *Acer mono* Maxim L. were collected from Changbai Mountain Jilin province, China. Seeds were exposed to cold stratification for six months from November to April. In early May, stratified seeds were sown once an open field nursery. After germination, seedlings were not treated with any kind of fertilizations until starting the experiment. After four-year uniform size, seedlings were selected in the same nursery, with plant-to-plant and row-to-row distances kept at 20 and 30 cm, respectively. Each seedling was treated with one of four levels of N (0, 5, 10, or 15 g) and one of four levels of P (0, 4, 6, or 8 g). Ten plants were included in each treatment, and each treatment was replicated three times (total n = 480). We applied N in two split doses during May and July, and P was applied once as a basal dose in May. Standard cultural practices were performed during the experiment (i.e., weeding, hoeing, irrigation, etc.), in order to produce healthy seedlings.

### Plant growth measurement

Plant height and root collar diameter were recorded for all selected seedlings before fertilizer application (May) and again at the end of the experiment in (November), in order to quantify plant growth during the experimental period. A digital venire caliper was used for the root collar diameter measurements (Shanghai measuring and cutting tools work. Co. Ltd).

### Estimation of chlorophyll contents and carotene

The chlorophylls a and b and carotene were measured, as described by Arnon [32]. Briefly, fresh leaves (0.2 g) were ground in a mortar with a small amount of quartz sand and calcium carbonate powder and 2–3 mL 95% ethanol. Absorbance of the supernatant was measured at 665, 649, and 470 nm using a spectrophotometer (Hitachi-U2001; Hitachi, Tokyo, Japan), and concentrations of chlorophylls a (*C*_a_) and b (*C*_b_) and carotene (*C*_x-c_) were calculated using the following formulae:

$$C_{a}=13.95A_{665}-6.88A_{649}\times \frac{VW}{1000}$$

$$C_{b}=24.96A_{649}-24.96A_{665}\times \frac{VW}{1000}$$

$$C_{x-c}=\frac{1000A_{470}-2.05C_{a}-114.8C_{b}}{245}\times \frac{VW}{1000}$$

where *A*_*i*_ indicates absorption at wavelength *i*, *V* is the volume of the extract, and *W* is the weight of fresh leaf tissue (g).

### Root morphology measurement

Root samples were carefully taken from single plant in each treatment group during harvesting, using the procedure described by Guo *et al*. [30]. The root samples were kept in an icebox and transported to the laboratory within 4 h of collection. The individual samples were cleaned with de-ionized water to remove residual soil particles and stored in a refrigerator. The root samples of each treatment were then divided into different branch orders, as described by Pregitzer *et al*. [33], i.e., the distal branch order as the first order. The separated samples were then scanned with an Expression 10000XL 1.0 scanner (dpi = 400; Epson Telford, Ltd., Telford, UK), and the images were analyzed using the WinRHIZO (Pro2004b) software (Instruments Regent Co., Ville de Québec, QC, Canada) to determine the average root diameter (ARD) and total root length (TRL). Finally, the roots were oven dried to constant mass at 65°C, in order to determine dry mass, and SRL was calculated as the TRL from each root order divided by the corresponding dry mass [34].

### Statistical analysis

The experiment was conducted with a randomized complete block design, with split plot arrangements, in order to test the effects of N and P fertilizer and their interaction on seedling growth, chlorophyll and carotene content, and root morphology. The data were analyzed using Statistix 8.1 software (Analytical Software, Tallahassee, FL, USA), and the experimental treatments were randomized and repeated three times, in order to reduce any variation caused by soil heterogeneity [35]. Furthermore, N treatment was used as the main block, whereas P treatment was used as the sub-block.

## Results

### Effect of N and P on plant growth

**Fig 1:**
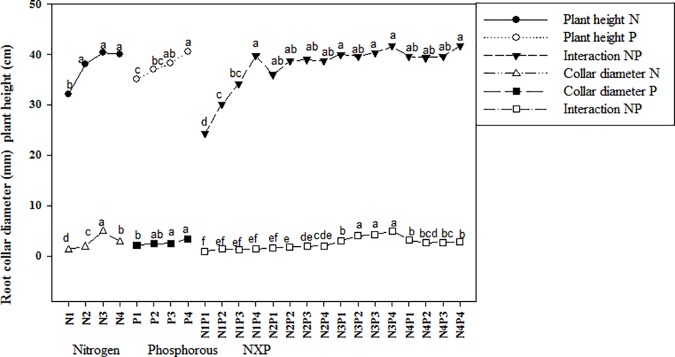
Combined and single effect of NP on plant height and collar diameter of Acer mono seedling. Different letters show the level of significance. Abbreviation N, Nitrogen; P, Phosphorous; NxP; Interaction.

Seedlings treated with N fertilizer exhibited significantly greater plant height and root diameter than untreated (i.e., 0 g N) controls (P<0.05; S1 Table, Fig 1), and values for both parameters were highest in seedlings treated with 10 g N, followed by those for seedlings treated with 15 and 5 g N, respectively. Similarly, seedlings treated with P fertilizer exhibited significantly greater plant height and root collar diameter than untreated (i.e., 0 g P) controls, and values for both parameters were highest in seedlings treated with 8 g P, followed by those treated with 6 and 4 g, respectively. We also observed a significant interaction effect of N and P for both plant height and root collar diameter and found that values for both plant height and root diameter were highest in seedlings treated with 10 g N and 8 g P (Fig 1).

### Effect of N and P on chlorophyll and carotene contents

**Fig 2:**
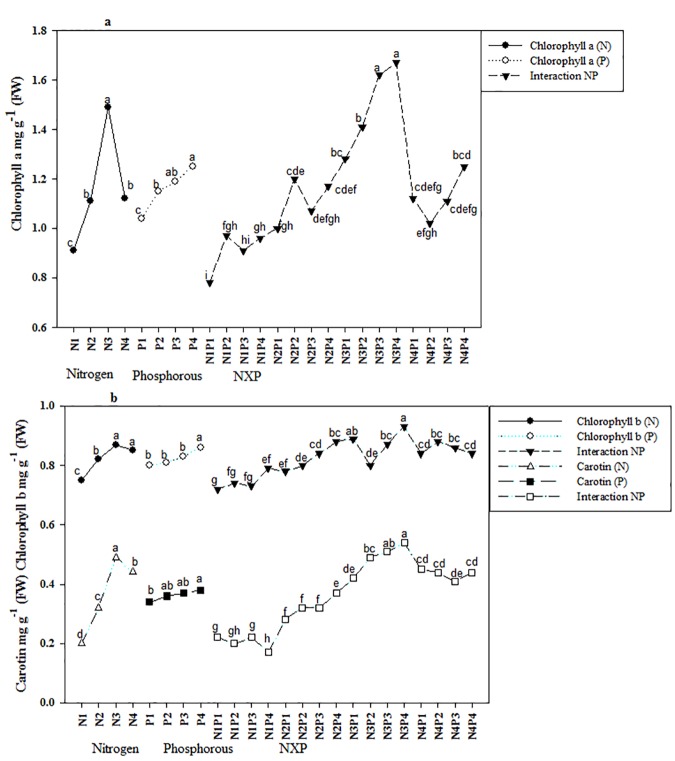
**a-b.** Combined and single effect of NP on chlorophylls a, b and carotene of Acer mono seedling. Different letters show the level of significance. Abbreviation N, Nitrogen; P, Phosphorous; NxP, Interaction.

Seedlings treated with N fertilizer exhibited significantly greater levels of chlorophylls a and b and carotene (P<0.05; S1 Table, Fig 2A and 2B), and values for all three parameters were greatest in seedlings treated with 10 g N. Similarly, seedlings treated with 8 g P fertilizer exhibited significantly greater levels of chlorophyll and carotene contents, whereas the values of seedlings treated with 6 and 4 g P were not significantly different than the values of plants treated with 0 g P. We also observed a significant interaction effect of N and P for levels of chlorophylls a and b and carotene and found that levels of all three were highest in seedlings treated with 10 g N and 8 g P (Fig 2A and 2B).

### Effect of N and P on root morphology

**Fig 3:**
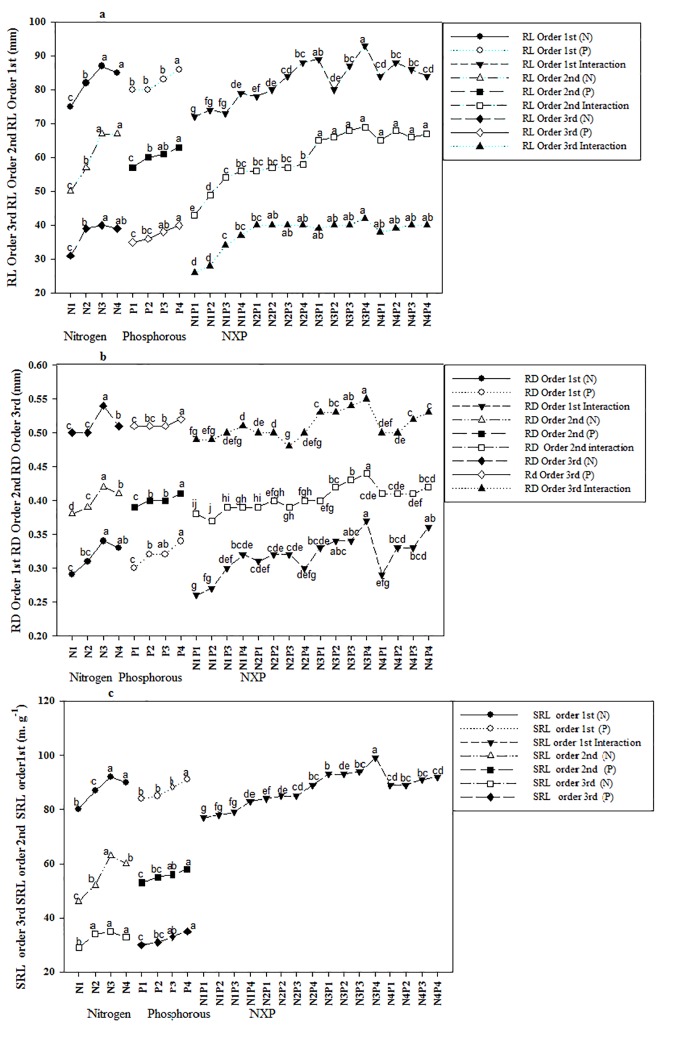
**a-c.** Combined and single effect of NP on root length, root diameter, and specific root length (SRL) of first, second and third order root of Acer mono seedling. Interaction values of specific root length (SRL) of second and third order root were non significant. Different letters show the level of significance. Abbreviation N, Nitrogen; P, Phosphorous; NxP, Interaction; RL, root length.

The first, second, and third root orders of seedlings treated with N fertilizer exhibited significantly greater TRL, ARD, and SRL values than those of untreated (i.e., 0 g N) seedlings (P<0.05; S1 Table, Fig 3A–3C), and for all root orders, values for the three parameters were highest in seedlings treated with 10 g N. The TRL, ARD, and SRL values varied among each of the root orders, depending on N level. Furthermore, both TRL and SRL decreased with increasing in root order but increased with increasing N level; whereas ARD increased with increasing root order and increased further with increasing N level.

Similarly, the first, second, and third root orders of seedlings treated with P fertilizer exhibited significantly greater TRL, ARD, and SRL values than those of untreated (i.e., 0 g P) seedlings (P<0.05; S1 Table, Fig 3A–3C). The root morphology values (i.e., TRL, ARD, and SRL) increases with increasing P level, and for all root orders, values for the three parameters were highest in seedlings treated with 8 g P. Furthermore, both TRL and SRL decreased with increasing in root order but increased with increasing P level; whereas ARD increased with increasing root order and increased further with increasing P level.

We also observed a significant interaction effect of N and P for TRL and ARD but found that interaction effect was only non significant for the SRL values of second and third order roots (P < 0.005; S1 Table). The TRL and ARD values of all three orders, as well as the SRL values of first order roots, were highest in seedlings treated with 10 g N and 8 g P (Fig 3A–3C).

## Discussion

### Fertilization and plant growth

Previous studies have shown that N supplementation can significantly affect the shoot morphology and nutritional status of nursery seedlings [36,37]. The growth-promoting effect of N (up to the optimum level) increases cytokinin production, which subsequently affects cell wall elasticity [38], number of meristematic cells, and cell growth [39]. In addition, N fertilization also increases seedling height and root collar diameter [40,41]. The present study demonstrated that N supplementation can increase growth parameters to a certain extent but has a negative effect at higher levels. Researchers have reported both positive and negative effects of fertilizer application on subsequent seedling growth and survival [42,43].

Meanwhile, P fertilizer application is necessary to ensure optimum plant production and quality [15], as well as for the acquisition, storage, and use of energy [16]. The present study demonstrated the positive relationship between P level and plant growth, which is supported by previous findings that P application increases plant height and root collar diameter [44], as well as basal stem diameter [45], and that P application has a positive effect on the growth of various species, including *Tectona grandis* [46], *Casuarina* spp. [47], *Dalbergia sissoo* [48], and *Tecoma grandis* [49]. The growth-promoting role of P application has been reported previously [50,51].

Furthermore, previous studies have also shown that root and shoot morphology are affected by the level and form of the fertilizer applied [52] and that the application of N and P fertilizer, either alone or in combination, significantly increases the stem growth of hybrid poplar seedlings [53], and combined P and N fertilization has been shown to promote the growth of young *Eucalyptus grandis* and birch (*Betula pubescens*) seedlings [54]. Therefore, the increased growth observed in *A*. *mono* seedlings treated with the optimal P level might have resulted from a favorable balance of nutrients (i.e., N and P).

### Fertilization and chlorophyll and carotene contents

The addition of N promotes the formation of active photosynthetic pigments by increasing the amounts of stromal and thylakoid proteins in leaves [55,56], as well as by increasing the formation of chloroplasts during leaf growth [57]. Although N is the most important elemental factor in chlorophyll biosynthesis, N application is also capable of producing negative effects [58]. For example, excess N has been shown to shorten the life span of leaves, increase their susceptibility to disease [59,60]. Chlorophyll and carotenoid synthesis are dependent upon mineral nutrition [61]. It might be due to the optimum availability of N, which plays a vital role in cell division and the formation of active photosynthetic pigments, including chlorophyll. Green pigments in leaves depend on P concentration, since it facilitates the plant for stability in unfavorable condition [62]. However, the facilitation of biochemical characteristics [51] and biosynthesis of pigment molecules depends on the uptake of optimal P levels [63]. In apricot seedlings, optimal P conditions have been shown to increase total chlorophyll content and plant growth [64]. Previous studies have also reported that P application increases the biomass and carotenoid production of a blue-green alga (*Spirulina platensis*) [65], whereas higher P concentrations were reported to reduce the chlorophyll content of the blue-green alga (*Azolla pinnata*) [66]. However, P deficiency decreases protein and chlorophyll content [67].

### Fertilization and root morphology

In the present study, optimum N fertilization had a favorable impact on root growth, which is supported by previous findings that N availability has significant effects on root biomass, production, and mortality [68]; root elongation [17]; and higher root-order development and branching [69]. The application of N fertilizer can also affect water use efficiency by influencing root growth and distribution in the soil [70], and optimal N fertilization has been shown to enhance root length and diameter [12], whereas higher and lower N levels have been shown to reduce root growth and biomass [71,72,73]. In addition, N fertilization significantly increases the diameter of *Larix gmelinii* root tips [74] and the growth, root length, and root diameter of *Pongamia pinnata* seedlings [75].

Furthermore, thin-root species have been shown to exhibit greater plasticity in response to fertilization, both in root growth rate and reducing mycorrhizal colonization [76]. Greater plasticity was also observed in thin-root species than in thick-root species in a subtropical forest [77]. High N levels have been shown to increase fine-root length and surface area in loblolly (*Pinus taeda* L.) and ponderosa pine *Pinus ponderosa* L. seedlings [78], which may potentially enhance nutrient and water acquisition from the soil. Plants exposed to high nutrient deficiencies exhibited a progressive reduction in TRL [23]. Therefore, N deficiency can result in a remarkable decrease in length of both first- and second-order lateral roots [79], a relationship that has been widely confirmed in both annual [71] and perennial plants [72].

In contrast, N availability has no significant effect on fine root biomass, SRL, ARD, or TRL [33,80]. For example, N fertilization increased the ARD of larch (*Larix gmelinii*) but had no effect on TRL [81] and was shown to enhance all three parameters (i.e., SRL, ARD, and TRL) in other tree species [82]. Furthermore, N is likely to have complementary effects on root morphology development because it stimulates root elongation [83]. The results of the present study are confirmed by previous findings that N fertilization increases SRL, up to a certain level [74,81], and SRL has also been associated with rapid root proliferation [84].

Previous studies have also shown that P can have a profound effect on root system morphology and that P generally stimulates root growth [85]. For example, P application significantly increases ARD and TRL in the perennial leguminous shrub *Lespedeza davurica* L. and the perennial herbaceous grass *Bothriochloa ischaemum* L. [20]. The present study also confirmed the results of Jin *et al*. [18], who reported that P application increases TRL and ARD. In most species, P-deficiency results in decreased ARD [86]; however, some species, such as *Arabidopsis thaliana*, develop larger roots in P-deficient conditions [87]. The SRL of fine roots varies, depending on nutrient availability [88]. Longer fine roots are more efficient in nutrient acquisition than shorter fine roots and allow plants to form larger root systems at a lower carbon cost than those of plants with shorter roots; whereas thicker roots may provide benefits in infertile or competitive environments [89]. Therefore, higher SRL values (i.e., thinner roots) indicate higher exploitation efficiency under intensive competition conditions, since plants with thinner roots are generally more competitive [90].

## Conclusions

The present study demonstrates that the growth and root morphology of *A*. *mono* seedlings are significantly affected by N and P fertilization. Untreated seedlings exhibited lower measures of plant height, root collar diameter, chlorophyll and carotene content, and several parameters of root morphology; whereas maximum values were achieved when seedlings were supplemented with optimal levels of N (10 g plant^-1^) and P (8 g plant^-1^). Therefore, the results of the present study suggest that optimal levels of N and P can be used to ensure the production of vigorous and healthy *A*. *mono* seedlings.

## Supporting information

S1 Table Summary of ANOVAs (F&P values) for the effect of fertilization on plant height, root collar diameter, chlorophyll and carotene content and root morphology of A. *mono* seedling. (DOCX)
