# Supplementary material for: Influence of nitrogen and phosphorous on the growth and root morphology of Acer mono
Source: PLoS One. 2017 Feb 24;12(2):e0171321. doi: 10.1371/journal.pone.0171321 (PMC5325205; doi:10.1371/journal.pone.0171321)
Supplement: S1 Table — (DOCX) [file pone.0171321.s001.docx]

# Supporting Information

**S1 Table**: Summary of ANOVAs (F&P values) for the effect of fertilization on plant height, root collar diameter, chlorophyll and carotene content and root morphology of A. *mono* seedling.

| **Effect** | | | **Plant height** | | | | | **Collar diameter** | | | | | | **Chlorophyll a** | | | | | | **Chlorophyll b** | | | | | **Carotene** | | | | |
| --- | --- | --- | --- | --- | --- | --- | --- | --- | --- | --- | --- | --- | --- | --- | --- | --- | --- | --- | --- | --- | --- | --- | --- | --- | --- | --- | --- | --- | --- |
|  | DF | | F | | | P | | F | | | P | | | F | | | P | | | F | | | P | | F | | | P | |
| **N** | 3 | | 14.8 | | | **0.0035** | | 68.01 | | | **0.0001** | | | 50.22 | | | **0.0001** | | | 41.91 | | | **0.0002** | | 197.29 | | | **0.0000** | |
| **P** | 3 | | 6.48 | | | **0.0032** | | 3.66 | | | **0.0265** | | | 7.71 | | | **0.0009** | | | 10.74 | | | **0.0001** | | 3.63 | | | **0.0273** | |
| **NxP** | 9 | | 2.62 | | | **0.0290** | | 2.51 | | | **0.0348** | | | 2.41 | | | **0.0410** | | | 5.24 | | | **0.0005** | | 5.29 | | | **0.0005** | |
| **Root length Root diameter** | | | | | | | | | | | | | | | | | | | | | | | | | | | | | |
|  | | | **1^st^ order 2^nd^ order 3^rd^ order** | | | | | | | | | | | | | **1^st^ order 2^nd^ order 3^rd^ order** | | | | | | | | | | | | | |
|  | DF | | F | | P | | F | | | P | | F | | | P | F | | | P | | F | | | P | | F | | | P |
| **N** | 3 | | 43.22 | | **0.0002** | | 172.28 | | | **0.0000** | | 250.28 | | | **0.0000** | 9.84 | | | **0.0098** | | 61.7 | | | **0.0001** | | 48.48 | | | **0.0001** |
| **P** | 3 | | 11.14 | | **0.0001** | | 8.57 | | | **0.0005** | | 9.1 | | | **0.0003** | 8.35 | | | **0.0006** | | 10.34 | | | **0.0001** | | 6.98 | | | **0.0015** |
| **NxP** | 9 | | 5.31 | | **0.0005** | | 2.71 | | | **0.0247** | | 2.68 | | | **0.0260** | 2.39 | | | **0.0425** | | 3.01 | | | **0.0150** | | 4.75 | | | **0.0011** |
| **Specific root length (SRL)** | | | | | | | | | | | | | | | | | | | | | | | | | | | | | |
|  | | | | **1^st^ order** | | | | | | | | | **2^nd^ order** | | | | | | | | | **3^rd^ order** | | | | | | | |
|  | | DF | | F | | | | | P | | | | F | | | | | P | | | | F | | | | | P | | |
| **N** | | 3 | | 56.62 | | | | | **0.0001** | | | | 131.93 | | | | | **0.0000** | | | | 149.01 | | | | | **0.0000** | | |
| **P** | | 3 | | 12.24 | | | | | **0.0002** | | | | 7.78 | | | | | **0.0008** | | | | 7.48 | | | | | **0.0011** | | |
| **NxP** | | 9 | | 5.55 | | | | | **0.0004** | | | | 2.1 | | | | | NS | | | | 2.15 | | | | | NS | | |

Note: Statistically significant probabilities (P < 0.05) are indicated in bold. NS indicates non-significant P-values.
